# Association of race/ethnicity and insurance with survival in patients with diffuse large B‐cell lymphoma in a large real‐world cohort

**DOI:** 10.1002/cam4.70032

**Published:** 2024-08-23

**Authors:** Yanling Jin, Jia Li, Yong Mun, Anthony Masaquel, Sylvia Hu, Juliana M. L. Biondo

**Affiliations:** ^1^ F. Hoffmann‐La Roche Ltd Mississauga Ontario Canada; ^2^ Genentech, Inc. South San Francisco California USA; ^3^ Present address: Data Solutions LLC Bronx New York USA

**Keywords:** diffuse large B‐cell lymphoma, Medicaid insurance status, overall survival, time to second‐line therapy or death

## Abstract

**Objective:**

Few studies have evaluated disparities in race, ethnicity, and health insurance in real‐world health outcomes for patients with diffuse large B‐cell lymphoma (DLBCL). This study aimed to evaluate association between racial disparities and health insurance with real‐world health outcomes.

**Methods:**

Patients with DLBCL (January 2011–July 2021) treated with first‐line therapy were selected from a real‐world database. Variables of interest included race/ethnicity, health insurance type (Medicaid, Commercial) by patient age (<65, ≥65 years), stage at diagnosis, overall survival (OS), and time to second‐line therapy or death due to any cause (TTNTD).

**Results:**

Among 5362 patients with DLBCL (82% White, 7% Black, 8% Hispanic/Latino, 3% Asian), White patients were older (mean age, 66.7 vs. 59.3–62.5 years) and less likely to have Medicaid insurance (1.7% vs. 3.4%–5.9%). Adjusted hazard ratios (aHR) for OS (Black, 0.88 [95% confidence interval, 0.72–1.07]; Hispanic/Latino, 0.84 [0.70–1.03]; Asian, 0.82 [0.59–1.16]) and TTNTD (Black, 0.89 [0.75–1.05]; Hispanic/Latino, 0.85 [0.73–1.00]; Asian, 1.11 [0.86–1.43]) were similar to those of White patients. Among patients aged <65 years, Medicaid‐insured versus Commercially insured patients had more advanced disease (stage III–IV, 66% vs. 48%), worse OS (aHR, 0.52 [0.34–0.80]; *p* = 0.003), and shorter TTNTD (aHR, 0.70 [0.49–0.99]; *p* = 0.044).

**Conclusions:**

There was no statistically significant difference in these variables/outcomes between Medicaid‐insured and commercially insured patients aged ≥65 years. Medicaid‐insured status was significantly associated with poorer OS and TTNTD in patients with DLBCL aged <65 years but not in those aged ≥65 years, with or without adjusting for other baseline characteristics. Race was not significantly associated with these outcomes.

The large real‐world EHR dataset Flatiron has shown that race was not significantly associated with poorer survival in patients with DLBCL. Medicaid insurance status was significantly associated with poorer overall survival and time to second‐line therapy or death due to any cause in patients with DLBCL aged <65 years.

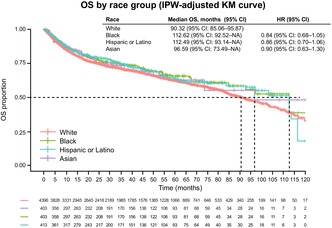

## 1 INTRODUCTION

Diffuse large B‐cell lymphoma (DLBCL) is the most common subtype of non‐Hodgkin lymphoma (NHL), accounting for approximately 30% of all NHL diagnoses.^1^ DLBCL is a highly heterogenous, aggressive hematological malignancy typically presenting with rapidly enlarging lymphadenopathy and constitutional symptoms, requiring urgent treatment.^2^ More than 18,000 patients are diagnosed with DLBCL each year in the United States (predominantly males), with a 5‐year relative survival rate of approximately 64%.^3^, ^4^

Although first‐line treatment comprising cyclophosphamide, doxorubicin, vincristine and prednisone and the anti‐CD20 monoclonal antibody rituximab (R‐CHOP) can achieve complete remission for many patients, over one‐third of patients with DLBCL do not respond to, or relapse after, R‐CHOP.^5^, ^6^, ^7^ For these patients with refractory or relapsed disease, alternative treatment options including salvage chemotherapy, autologous stem cell transplant or alternative immunotherapeutic approaches are used, but outcomes are often poor.^8^, ^9^

Disease stage and treatment are widely acknowledged as key factors affecting health outcomes for cancer patients.^10^ However, in addition to disease stage and treatment, disparities in health outcomes due to race, ethnicity, and socioeconomic status are also documented in the literature, with certain groups at increased risk of developing or succumbing to particular cancers.^11^ An example of this is the documented higher mortality rate for Black patients compared with all other racial/ethnic groups for many, although not all, cancer types.^12^ Furthermore, individuals who live in USA counties that experience persistent poverty are more likely to die from cancer than those who live in more wealthy counties.^13^ Male gender has also been associated with worse survival among patients with DLBCL treated with R‐CHOP.^14^

A small number of studies have specifically addressed socioeconomic disparities, using insurance as a proxy, or racial/ethnic disparities in treatment patterns and health outcomes for patients with DLBCL.^15^, ^16^, ^17^, ^18^, ^19^ Though some of the above studies used large datasets such as the Surveillance, Epidemiology, and End Results (SEER)^16^, ^17^ and the National Cancer Database (NCDB),^15^ the Flatiron Health database provides an opportunity to study the effect of race and insurance status on cancer survival in a large population of patients with DLBCL in the USA. Flatiron Health includes clinical and outcome data unique among real‐world datasets, allowing detailed investigation including sequential time‐to‐event endpoints.^20^ To our knowledge, no studies have addressed in detail disparities in both health insurance access and race/ethnicity and the relationship with health outcomes for patients with DLBCL based on recent data. Here, we leverage the Flatiron Health database to evaluate the association of race, ethnicity, and health insurance status with treatment outcomes in patients with DLBCL. Based on published findings, we hypothesize that insurance status and race will be significantly linked to poorer survival outcomes in patients with DLBCL.

## 2 METHODS

### 2.1 Data source

We utilized the Flatiron Health database, a large, longitudinal, nationwide USA oncology de‐identified electronic health record‐derived database that can be used for tumor‐specific analyses. During the study period, the de‐identified data originated from approximately 280 USA cancer clinics (approximately 800 sites of care). The database comprises de‐identified patient‐level structured and unstructured data and is curated via technology‐enabled abstraction^20^, ^21^ which aims to increase the accuracy and efficiency with which human abstractors can extract critical data elements from patient charts. Institutional Review Board approval of the study protocol was obtained prior to study conduct and included a waiver of informed consent.

### 2.2 Study population

Patients diagnosed with DLBCL (using the International Classification of Diseases [ICD] code‐9 [200x, 202x] and ICD‐10 [C82x, C83x, C84x, C85x, C86x, C88x, C96x] for NHL) from January 2011 to July 2021 and treated with first‐line therapy within 90 days of diagnosis were selected from the database. The reason for applying the 90‐day rule was to reduce the chance of misclassifying later lines of therapy as the first‐line therapy due to missing treatment data. Patients had at least two documented clinical visits, on different days, occurring on or after 1 January 2011. Patients of “other” race (i.e., American Indian or Alaska Native, Hawaiian or Pacific Islander, multiple race) or who had missing data were excluded.

### 2.3 Study variables

Variables of interest included demographic variables (i.e., age, sex, race/ethnicity, patients' geographic location), clinical characteristics (i.e., Eastern Cooperative Oncology Group [ECOG] performance status, disease stage, transformation status, extranodal disease, cell of origin), site of care (Academic vs. Community), biomarker information (*BCL2, BCL6, CD30, EBER*, MYC, lactate dehydrogenase [LDH]), and health insurance type. Based on the combination of race and ethnicity reported in the Flatiron Health database, patients were categorized into the following race/ethnicity groups: Non‐Hispanic White (White), Non‐Hispanic Black (Black), Hispanic or Latino, and Non‐Hispanic Asian (Asian). For the Hispanic or Latino cohort, race was classed as Hispanic or Latino if a patient's corresponding ethnicity was Hispanic or Latino.

Health insurance was categorized according to the following types: Medicaid without Commercial (Medicaid), Commercial without Medicaid (Commercial), and “other” (for remaining patients, including Commercial and Medicaid insurance concurrently, no Commercial and Medicaid insurance, and missing data). For insurance analyses, only valid records (i.e., where the insurance coverage period included the patient's diagnosis date) were included.

Outcome variables included the following time‐to‐event endpoints: the primary endpoint of OS (i.e., the time from start of first‐line treatment to death or censor at last alive date), and the secondary endpoints of time to second‐line therapy (used as a proxy for progression‐free survival [PFS], as data on PFS were not available in the Flatiron Health database for patients with DLBCL) and death due to any cause (i.e., time from the start of first‐line treatment to death or start of second‐line therapy [TTNTD]).

### 2.4 Statistical analysis

Baseline characteristics, OS, and TTNTD were compared between race groups as follows White versus Black versus Hispanic or Latino versus Asian patients. The same parameters were also compared between medical insurance groups including Medicaid (USA government program that is typically available to those with a lower income and/or disability) versus Commercial in patients aged <65 years and aged ≥65 years. Medicare (USA government program available to those aged ≥65 years, and those with specific chronic conditions) was assumed for all patients aged ≥65 years. Fisher's exact, chi‐squared, or *t*‐tests were used to compare baseline characteristics. Time‐to‐event endpoints (OS and TTNTD) were compared using Cox models, and inverse probability weighting (IPW) was used to adjust for differences in baseline characteristics. Kaplan–Meier analysis was used to estimate medians for time‐to‐event endpoints. Data were analyzed with R version 4.0.0 and are reported up to the cut‐off date of 31 July 2021.

## 3 RESULTS

### 3.1 Patients

**FIGURE 1:**
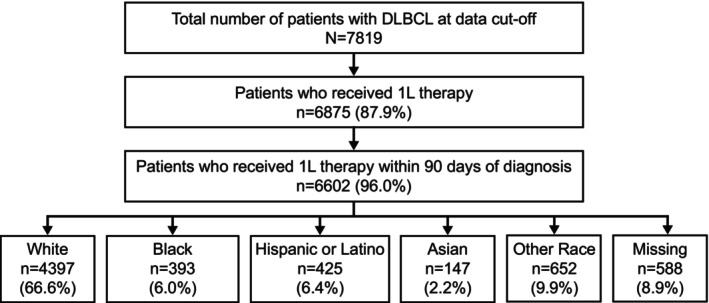
Patient disposition and study populations. 1 L, first‐line; DLBCL, diffuse large B‐cell lymphoma.

From January 2011 to July 2021, 7819 patients diagnosed with DLBCL were identified, including 6602 patients (84.4%) who received first‐line treatment. Of these 6602 patients, 4397 (66.6%) were White, 393 (6.0%) were Black, 425 (6.4%) were Hispanic or Latino, 147 (2.2%) were Asian, and 1240 (18.8%) were of “other” race or had missing data (Figure 1). In total, 5362 patients were included in the study population. This group had a median 46 months of follow up and included White (82.0%), Black (7.3%), Hispanic or Latino (7.9%), and Asian patients (2.7%).

Compared with other race groups, White patients were older (mean age 66.7 vs. 59.3–62.5 years) and were less likely to have Medicaid insurance (1.7% vs. 3.4–5.9%) (Table 1). White patients had the lowest Medicaid to Commercial insurance ratio at 0.05, compared with 0.16 for Black patients, 0.20 for Hispanic or Latinos, and 0.13 for Asians. Stage III–IV disease was more common among Black patients compared with other race groups (56.2% vs. 49.6% [White], 45.9% [Hispanic or Latino], and 46.9% [Asian]). Across race groups, patients' sites of care (Academic vs. Community) were similar.

**TABLE 1:** Patient disposition and baseline characteristics.

Characteristic	White (*n* = 4397)	Black (*n* = 393)	Hispanic or Latino (*n* = 425)	Asian (*n* = 147)	*p*
Mean age (SD)^a^, years	66.7 (12.6)	59.3 (15.1)	60.9 (15.2)	62.5 (15.9)	<0.001
Age, *n* (%)					
<65 years	1582 (36.0)	230 (58.5)	218 (51.3)	72 (49.0)	<0.001
≥65 years	2815 (64.0)	163 (41.5)	207 (48.7)	75 (51.0)	
Female sex, *n* (%)	1929 (43.9)	178 (45.3)	200 (47.1)	76 (51.7)	0.171
ECOG PS, *n* (%)					
0–1	1682 (38.3)	129 (32.8)	141 (33.2)	51 (34.7)	<0.001
≥2	327 (7.4)	21 (5.3)	17 (4.0)	6 (4.1)	
Missing	2388 (54.3)	243 (61.8)	267 (62.8)	90 (61.2)	
Group stage status^b^ *n* (%)					
I–II	1276 (29.0)	85 (21.6)	124 (29.2)	47 (32.0)	0.022
III–IV	2181 (49.6)	221 (56.2)	195 (45.9)	69 (46.9)	
Missing	940 (21.4)	87 (22.1)	106 (24.9)	31 (21.1)	
Transformed from a prior indolent lymphoid malignancy, *n* (%)					
Yes	667 (15.2)	62 (15.8)	41 (9.6)	13 (8.8)	0.003
No	3730 (84.8)	331 (84.2)	384 (90.4)	134 (91.2)	
Extranodal disease at diagnosis, *n* (%)					
Yes	2431 (55.3)	223 (56.7)	223 (52.5)	96 (65.3)	0.055
No	1966 (44.7)	170 (43.3)	202 (47.5)	51 (34.7)	
Cell of origin^b^, *n* (%)					
GCB	1406 (32.0)	114 (29.0)	110 (25.9)	46 (31.3)	<0.001
ABC	935 (21.3)	83 (21.1)	117 (27.5)	50 (34.0)	
Unknown/undocumented	2056 (46.8)	196 (49.9)	198 (46.6)	51 (34.7)	
Geographic location, *n* (%)					
North‐East	631 (14.4)	44 (11.2)	60 (14.1)	19 (12.9)	<0.001
South	1818 (41.3)	240 (61.1)	137 (32.2)	28 (19.0)	
West	497 (11.3)	14 (3.6)	122 (28.7)	72 (49.0)	
Unknown/others	1451 (33.0)	95 (24.2)	106 (24.9)	28 (19.0)	
Insurance type, *n* (%)					
Medicaid (without Commercial)	75 (1.7)	22 (5.6)	25 (5.9)	5 (3.4)	<0.001
Commercial (without Medicaid)	1445 (32.9)	136 (34.6)	126 (29.6)	40 (27.2)	
Ratio of Medicaid versus Commercial	0.05	0.16	0.20	0.13	
Other^c^	2877 (65.4)	235 (59.8)	274 (64.5)	102 (69.4)	
*BCL2*, *n* (%)					
Positive	2130 (48.4)	177 (45.0)	226 (53.2)	76 (51.7)	0.045
Negative	631 (14.4)	76 (19.3)	63 (14.8)	18 (12.2)	
Unknown	1636 (37.2)	140 (35.6)	136 (32.0)	53 (36.1)	
*BCL6*, *n* (%)					
Positive	2535 (57.7)	217 (55.2)	249 (58.6)	88 (59.9)	0.475
Negative	414 (9.4)	35 (8.9)	46 (10.8)	18 (12.2)	
Unknown	1448 (32.9)	141 (35.9)	130 (30.6)	41 (27.9)	
*CD30*, *n* (%)					
Positive	452 (10.3)	52 (13.2)	53 (12.5)	9 (6.1)	0.158
Negative	1327 (30.2)	121 (30.8)	132 (31.1)	50 (34.0)	
Unknown	2618 (59.5)	220 (56.0)	240 (56.5)	88 (59.9)	
*EBER*, *n* (%)					
Positive	38 (0.9)	5 (1.3)	2 (0.5)	2 (1.4)	0.149
Negative	493 (11.2)	52 (13.2)	58 (13.6)	25 (17.0)	
Unknown	3866 (87.9)	336 (85.5)	365 (85.9)	120 (81.6)	
MYC^d^, *n* (%)					
Positive	644 (14.6)	66 (16.8)	67 (15.8)	28 (19.0)	0.345
Negative	432 (9.8)	44 (11.2)	33 (7.8)	12 (8.2)	
Unknown	3321 (75.5)	283 (72.0)	325 (76.5)	107 (72.8)	
Elevated LDH, *n* (%)					
Yes	1862 (42.3)	156 (39.7)	136 (32.0)	43 (29.3)	<0.001
No	928 (21.1)	60 (15.3)	69 (16.2)	35 (23.8)	
Unknown	1607 (36.5)	177 (45.0)	220 (51.8)	69 (46.9)	
Site of care					
Academic	680 (15.5)	66 (16.8)	49 (11.5)	22 (15.0)	0.142
Community	3717 (84.5)	327 (83.2)	376 (88.5)	125 (85.0)	

*Note*: All data are *n* (%) unless otherwise specified. *p*‐values calculated as White patients versus other patient groups.

Abbreviations: ABC, non‐germinal B cell/activated B cell; ECOG PS, Eastern Cooperative Oncology Group performance status; GCB, germinal center B cell; IHC, immunohistochemistry; LDH, lactate dehydrogenase; SD, standard deviation.

^a^ At first treatment.

^b^ At initial diagnosis.

^c^ Includes Commercial and Medicaid, Medicare/Patient assistance program/government aid without Commercial or Medicaid, and missing.

^d^ By IHC.

Among patients aged <65 years, more Medicaid‐insured patients had advanced disease than Commercially insured patients (stage III–IV, 65.8% vs. 47.7%), whereas the difference was smaller in patients aged ≥65 years (59.3% vs. 49.8%) (Table 2).

**TABLE 2:** Patient disposition and baseline characteristics by insurance.

Characteristic	<65 years			≥65 years		
	Medicaid (without Commercial) (*n* = 73)	Commercial (without Medicaid) (*n* = 807)	*p*	Medicaid (without Commercial) (*n* = 54)	Commercial (without Medicaid) (*n* = 940)	*p*
Mean age (SD)^a^, years	51.2 (10.4)	52.3 (10.9)	0.386	73.2 (5.3)	73.9 (5.3)	0.308
Female sex, *n* (%)	29 (39.7)	351 (43.5)	0.618	30 (55.6)	414 (44.0)	0.130
ECOG PS, *n* (%)						
0–1	19 (26.0)	322 (39.9)	0.017	19 (35.2)	387 (41.2)	0.327
≥2	6 (8.2)	28 (3.5)		3 (5.6)	88 (9.4)	
Missing	48 (65.8)	457 (56.6)		32 (59.3)	465 (49.5)	
Disease stage^b^, *n* (%)						
I–II	12 (16.4)	271 (33.6)	0.005	12 (22.2)	257 (27.3)	0.400
III–IV	48 (65.8)	385 (47.7)		32 (59.3)	468 (49.8)	
Missing	13 (17.8)	151 (18.7)		10 (18.5)	215 (22.9)	
Transformed from a prior indolent lymphoid malignancy, *n* (%)						
Yes	6 (8.2)	105 (13.0)	0.319	3 (5.6)	139 (14.8)	0.092
No/unknown	67 (91.8)	702 (87.0)		51 (94.4)	801 (85.2)	
Extranodal disease at diagnosis, *n* (%)						
Yes	46 (63.0)	433 (53.7)	0.157	29 (53.7)	552 (58.7)	0.558
No/unknown	27 (37.0)	374 (46.3)		25 (46.3)	388 (41.3)	
Cell of origin^b^, *n* (%)						
GCB	29 (39.7)	277 (34.3)	0.337	18 (33.3)	306 (32.6)	0.705
ABC	10 (13.7)	166 (20.6)		10 (18.5)	219 (23.3)	
Unknown/undocumented	34 (46.6)	364 (45.1)		26 (48.1)	415 (44.1)	
Geographic location, *n* (%)						
North‐East	5 (6.8)	101 (12.5)	0.356	7 (13.0)	169 (18.0)	0.024
South	31 (42.5)	354 (43.9)		33 (61.1)	413 (43.9)	
West	9 (12.3)	107 (13.3)		1 (1.9)	126 (13.4)	
Unknown/others	28 (38.4)	245 (30.4)		13 (24.1)	232 (24.7)	
Race, *n* (%)						
White	46 (63.0)	613 (76.0)	0.026	29 (53.7)	832 (88.5)	<0.001
Black	16 (21.9)	86 (10.7)		6 (11.1)	50 (5.3)	
Hispanic or Latino	9 (12.3)	81 (10.0)		16 (29.6)	45 (4.8)	
Asian	2 (2.7)	27 (3.3)		3 (5.6)	13 (1.4)	
*BCL2*, *n* (%)						
Positive	31 (42.5)	375 (46.5)	0.650	29 (53.7)	486 (51.7)	0.926
Negative	13 (17.8)	155 (19.2)		7 (13.0)	116 (12.3)	
Unknown	29 (39.7)	277 (34.3)		18 (33.3)	338 (36.0)	
*BCL6*, *n* (%)						
Positive	44 (60.3)	491 (60.8)	0.993	32 (59.3)	546 (58.1)	0.776
Negative	6 (8.2)	67 (8.3)		7 (13.0)	99 (10.5)	
Unknown	23 (31.5)	249 (30.9)		15 (27.8)	295 (31.4)	
*CD30*, *n* (%)						
Positive	5 (6.8)	120 (14.9)	0.068	3 (5.6)	71 (7.6)	0.778
Negative	20 (27.4)	257 (31.8)		19 (35.2)	297 (31.6)	
Unknown	48 (65.8)	430 (53.3)		32 (59.3)	572 (60.9)	
*EBER*, *n* (%)						
Positive	0 (0.0)	4 (0.5)	0.774	0 (0.0)	9 (1.0)	0.434
Negative	9 (12.3)	112 (13.9)		7 (13.0)	81 (8.6)	
Unknown	64 (87.7)	691 (85.6)		47 (87.0)	850 (90.4)	
MYC^c^, *n* (%)						
Positive	14 (19.2)	122 (15.1)	0.400	7 (13.0)	158 (16.8)	0.760
Negative	6 (8.2)	103 (12.8)		5 (9.3)	81 (8.6)	
Unknown	53 (72.6)	582 (72.1)		42 (77.8)	701 (74.6)	
Elevated LDH, *n* (%)						
No	10 (13.7)	172 (21.3)	0.049	11 (20.4)	206 (21.9)	0.957
Yes	27 (37.0)	349 (43.2)		24 (44.4)	417 (44.4)	
Unknown	36 (49.3)	286 (35.4)		19 (35.2)	317 (33.7)	
Site of care, *n* (%)						
Academic	16 (21.9)	131 (16.2)	0.279	5 (9.3)	75 (8.0)	0.937
Community	57 (78.1)	676 (83.8)		49 (90.7)	865 (92.0)	

*Note*: Data are *n* (%) unless otherwise specified.

Abbreviations: ABC, non‐germinal B cell/activated B cell; ECOG PS, Eastern Cooperative Oncology Group performance status; GCB, germinal center B cell; IHC, immunohistochemistry; LDH, lactate dehydrogenase; SD, standard deviation.

^a^ At first treatment.

^b^ At initial diagnosis.

^c^ By IHC.

#### 3.1.1 Treatment within versus after 90 days

The analysis cohort excluded patients who were treated after 90 days (4% of patients who had received first‐line therapy [Figure 1]) in order to reduce the chance that the lines of therapy were misclassified. The distribution of patients who were treated within 90 days was similar among the different race groups (94.2%–96.4%) and insurance groups (97.1%–98.4%) (Table S1).

### 3.2 Medical insurance by age

In patients aged <65 years, 38.4% had Commercial insurance alone, 3.5% had Medicaid insurance alone, 1.3% had Commercial and Medicaid insurance concurrently, 21.4% had no Commercial or Medicaid insurance, and 35.4% had no insurance or missing (Table 3). For patients aged ≥65 years (for whom Medicare was assumed based on eligibility criteria), 28.8% had Commercial insurance alone, 1.7% had Medicaid insurance alone, 0.6% had Commercial and Medicaid insurance concurrently, and 69.0% had no Commercial or Medicaid insurance (Table 3).

**TABLE 3:** Medical insurance by age.

Insurance^a^, *n* (%)	Age < 65 years (*n* = 2102)	Age ≥ 65 years (*n* = 3260)
Commercial (without Medicaid)	807 (38.4)	940 (28.8)
Medicaid (without Commercial)	73 (3.5)	54 (1.7)
Other	1222 (58.1)	2266 (69.5)
Commercial and Medicaid concurrently	27 (1.3)	18 (0.6)
No commercial and Medicaid	450 (21.4)	2248 (69.0)
Medicare without patient assistance program or government aid	27 (1.3)	2060 (63.2)
Patient assistance program or government aid without Medicare	136 (6.5)	0
Medicare and patient assistance/government aid	5 (0.2)	188 (5.8)
Other payer—type unknown or self pay without medicare/patient assistance/government aid	282 (13.4)	0
No insurance or missing	745 (35.4)	0

*Note*: All data are *n* (%).

^a^ Insurance categories are based on the categories available in the Flatiron Health Insurance database.

### 3.3 Time‐to‐event endpoints

#### 3.3.1 Overall survival

**FIGURE 2:**
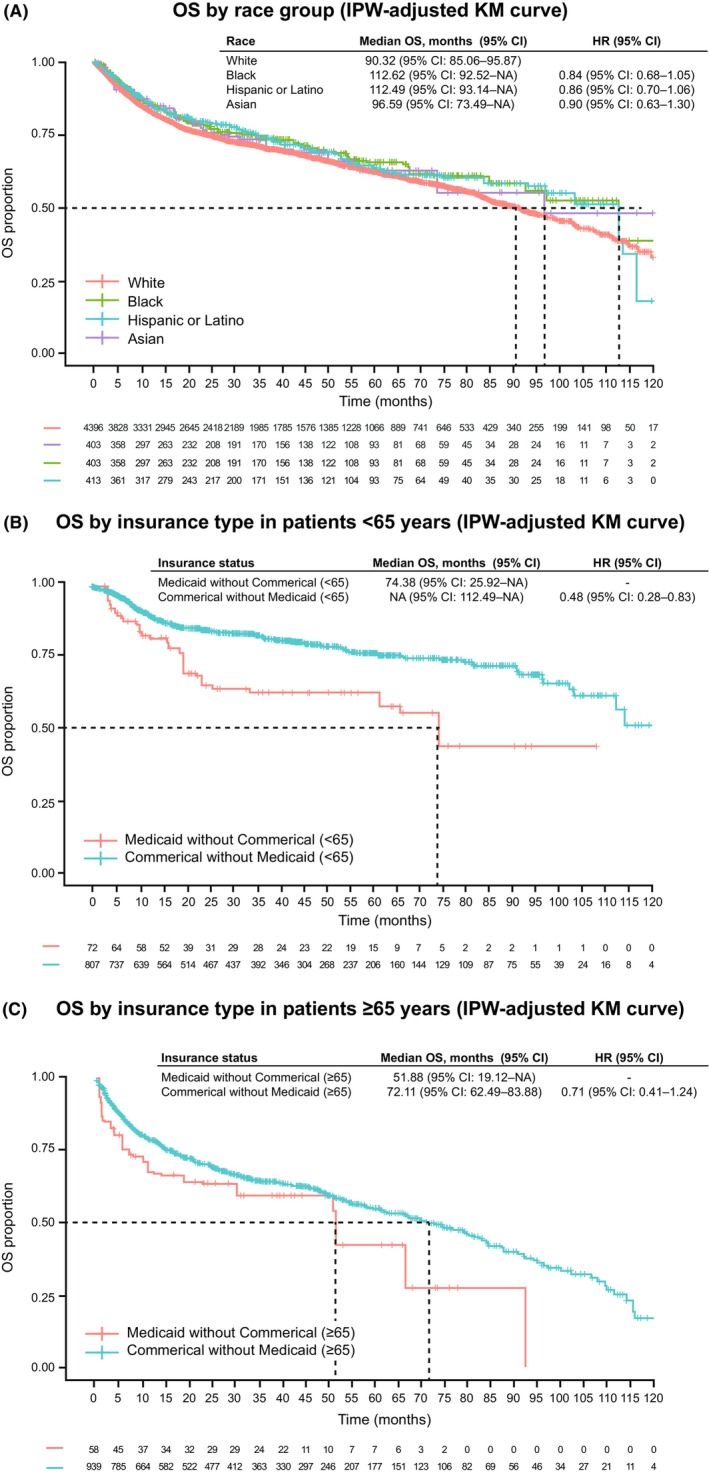
(A) OS from start of treatment (IPW‐adjusted KM curve) by race group. (B) OS by insurance type in patients aged <65 years. (C) OS by insurance type in patients aged ≥65 years. CI, confidence interval; HR, hazard ratio; IPW, inverse probability weighting; KM, Kaplan–Meier; NA, not available; OS, overall survival.

In the univariable analysis, patient age at first‐line treatment, sex, ECOG performance status at initial diagnosis, disease stage at initial diagnosis, extranodal disease status, cell of origin, *BCL2, BCL6, CD30*, MYC, LDH level, site of care, and insurance status were all statistically significantly associated with OS (Appendix S1; Table S2). After adjusting for these statistically significant variables from the univariable analysis, multivariable analyses based on the Cox model (with insurance by age variable; *p* = 0.159) and IPW analysis (using propensity score matching) did not show a statistically significant difference in OS across race groups. Hazard ratios (HRs) for comparisons with White patients were 0.88 (95% confidence interval [CI], 0.72–1.07) for Black patients, 0.84 (95% CI, 0.70–1.03) for Hispanic or Latino patients, and 0.82 (95% CI, 0.59–1.16) for Asian patients in multivariable analyses based on the Cox model (Table 4). Corresponding HRs for IPW analyses were 0.84 (95% CI, 0.68–1.05), 0.86 (95% CI, 0.70–1.06), and 0.90 (95% CI, 0.63–1.30) (Figure 2A). Median OS values by the IPW method were 112.6 months (95% CI, 92.5–Not Available [NA]) for Black patients, 112.5 months (95% CI, 93.1–NA) for Hispanic or Latino patients, 96.6 months (95% CI, 73.5–NA) for Asian patients, and 90.3 months (95% CI, 85.1–95.9) for White patients (Table 4; Figure 2A). For the unadjusted univariable analyses, see Figure S1A.

**TABLE 4:** Summary of time‐to‐event endpoints based on a Cox model (multivariable analysis).

Overall survival	Multivariable		
	HR (95% CI)	Pair‐wise *p*	Overall *p*
Race			
White (ref.) (*n* = 4397)			
Black (*n* = 393)	0.88 (0.72–1.07)	0.202	0.159
Hispanic or Latino (*n* = 425)	0.84 (0.70–1.03)	0.087	
Asian (*n* = 147)	0.82 (0.59–1.16)	0.267	
Age^a^			
<50 years (ref.) (*n* = 631)			
50–64 years (*n* = 1471)	1.75 (1.36–2.25)	< 0.001	<0.001
65–79 years (*n* = 2743)	1.75 (1.12–2.74)	0.015	
≥80 years (*n* = 517)	2.48 (1.55–3.95)	< 0.001	
Sex			
Female (ref.) (*n* = 2383)			
Male (*n* = 2979)	1.22 (1.11–1.34)	< 0.001	<0.001
ECOG PS			
0–1 (ref.) (*n* = 2003)			
≥2 (*n* = 371)	2.15 (1.83–2.53)	<0.001	<0.001
Missing (*n* = 2988)	1.12 (1.00–1.26)	0.053	
Disease stage^b^			
I–II (ref.) (*n* = 1532)			
III–IV (*n* = 2666)	1.51 (1.33–1.71)	<0.001	<0.001
Missing (*n* = 1164)	2.06 (1.79–2.37)	<0.001	
Extranodal disease at diagnosis			
Yes (ref.) (*n* = 2973)			
No/unknown (*n* = 2389)	1.14 (1.03–1.25)	0.009	0.009
Cell of origin^b^			
GCB (ref.) (*n* = 1676)			
ABC (*n* = 1185)	1.20 (1.04–1.37)	0.011	0.035
Unknown/undocumented (*n* = 2501)	1.06 (0.94–1.19)	0.375	
*BCL2*			
Positive (ref.) (*n* = 2609)			
Negative (*n* = 788)	0.81 (0.70–0.95)	0.009	0.012
Unknown (*n* = 1965)	0.88 (0.77–1.00)	0.045	
*BCL6*			
Positive (ref.) (*n* = 3089)			
Negative (*n* = 513)	1.18 (1.00–1.38)	0.050	0.047
Unknown (*n* = 1760)	1.13 (0.99–1.29)	0.060	
*CD30*			
Positive (ref.) (*n* = 566)			
Negative (*n* = 1630)	1.16 (0.97–1.40)	0.113	0.283
Unknown (*n* = 3166)	1.12 (0.94–1.33)	0.221	
MYC^c^			
Positive (ref.) (*n* = 805)			
Negative (*n* = 521)	0.77 (0.62–0.96)	0.022	0.043
Unknown (*n* = 4036)	0.86 (0.74–1.00)	0.044	
Elevated LDH			
No (ref.) (*n* = 1092)			
Yes (*n* = 2197)	1.38 (1.20–1.59)	< 0.001	<0.001
Unknown (*n* = 2073)	1.32 (1.14–1.53)	< 0.001	
Site of care			
Academic (ref.) (*n* = 817)			
Community (*n* = 4545)	1.22 (1.06–1.41)	0.007	0.007
Insurance type			
<65 years: Medicaid (without Commercial) (ref.) (*n* = 73)			
<65 years: Commercial (without Medicaid) (*n* = 807)	0.52 (0.34–0.80)	0.003	0.002
<65 years: Other (*n* = 1222)	0.48 (0.31–0.72)	< 0.001	
≥65 years: Medicaid (without Commercial) (ref.) (*n* = 54)			
≥65 years: Commercial (without Medicaid) (*n* = 940)	0.76 (0.50–1.17)	0.213	0.386
≥65 years: Other (*n* = 2266)	0.80 (0.53–1.22)	0.304	
**Time to second‐line therapy or death due to any cause**			
Race			
White (ref.) (*n* = 4393)			
Black (*n* = 393)	0.89 (0.75–1.05)	0.152	0.099
Hispanic or Latino (*n* = 425)	0.85 (0.73–1.00)	0.055	
Asian (*n* = 147)	1.11 (0.86–1.43)	0.418	
Age^a^			
<50 years (ref.) (*n* = 631)			
50–64 years (*n* = 1470)	1.21 (1.02–1.43)	0.027	0.002
65–79 years (*n* = 2740)	1.16 (0.81–1.66)	0.415	
≥80 years (*n* = 517)	1.45 (0.99–2.10)	0.054	
Sex			
Female (ref.) (*n* = 2380)			
Male (*n* = 2978)	1.16 (1.07–1.25)	<0.001	<0.001
ECOG PS			
0–1 (ref.) (*n* = 2002)			
≥2 (*n* = 371)	1.77 (1.53–2.05)	<0.001	<0.001
Missing (*n* = 2985)	1.05 (0.95–1.15)	0.363	
Disease stage^b^			
I–II (ref.) (*n* = 1531)			
III–IV (*n* = 2663)	1.54 (1.39–1.71)	<0.001	<0.001
Missing (*n* = 1164)	1.88 (1.67–2.12)	<0.001	
Transformed from a prior indolent lymphoid malignancy			
Yes (ref.) (*n* = 783)			
No/unknown (*n* = 4575)	1.23 (1.11–1.37)	<0.001	<0.001
Extranodal disease at diagnosis			
Yes (ref.) (*n* = 2970)			
No/unknown (*n* = 2388)	1.13 (1.04–1.22)	0.004	0.004
Cell of origin^b^			
GCB (ref.) (*n* = 1673)			
ABC (*n* = 1185)	1.11 (0.99–1.25)	0.076	0.138
Unknown/undocumented (*n* = 2500)	1.00 (0.91–1.11)	0.930	
*BCL2*			
Positive (ref.) (*n* = 2609)			
Negative (*n* = 787)	0.75 (0.66–0.85)	<0.001	<0.001
Unknown (*n* = 1932)	0.88 (0.79–0.97)	0.013	
*BCL6*			
Positive (ref.) (*n* = 3088)			
Negative (*n* = 513)	1.18 (1.03–1.35)	0.017	0.058
Unknown (*n* = 1757)	1.04 (0.93–1.16)	0.537	
Elevated LDH			
No (ref.) (*n* = 1092)			
Yes (*n* = 2194)	1.46 (1.30–1.65)	<0.001	<0.001
Unknown (*n* = 2072)	1.46 (1.29–1.65)	<0.001	
Site of care			
Academic (ref.) (*n* = 817)			
Community (*n* = 4541)	0.89 (0.80–1.00)	0.047	0.047
Insurance type			
<65 years: Medicaid (without Commercial) (ref.) (*n* = 73)			
<65 years: Commercial (without Medicaid) (*n* = 806)	0.70 (0.49–0.99)	0.044	0.041
<65 years: Other (*n* = 1222)	0.65 (0.46–0.92)	0.014	
≥65 years: Medicaid (without Commercial) (ref.) (*n* = 54)			
≥65 years: Commercial (without Medicaid) (*n* = 940)	0.77 (0.53–1.12)	0.172	0.390
≥65 years: Other (*n* = 2263)	0.79 (0.55–1.14)	0.205	

Note: Data are HR (95% CI). OS was defined as time from start of the first‐line treatment to death or censor at last alive date. TTNTD was defined as time from the start of the first‐line treatment. Please note, “ref.” refers to the reference group for the *p*‐value comparison for each variable.

Abbreviations: ABC, non‐germinal B cell/activated B cell; CI, confidence interval; ECOG PS, Eastern Cooperative Oncology Group performance status; GCB, germinal center B cell; HR, hazard ratio; LDH, lactate dehydrogenase; OS, overall survival; TTNTD, time to second‐line treatment or death.

^a^ At first treatment.

^b^ At initial diagnosis.

^c^ By IHC.

Significantly shorter OS among Medicaid‐insured patients aged <65 years was confirmed in the multivariable (HR, 0.52 [95% CI, 0.34–0.80]; *p* = 0.003) and IPW analyses (HR, 0.48 [95% CI, 0.28–0.83]; *p* = 0.008) (Table 4; Figure 2B). Median OS by IPW was 74.4 months (95% CI, 25.9–NA) for Medicaid‐insured patients and NA (95% CI, 112.5–NA) for Commercially insured patients (Figure 2B). For patients aged ≥65 years, multivariable analysis based on the Cox model (HR, 0.76 [95% CI, 0.50–1.17]; *p* = 0.213) and IPW analysis (HR, 0.71 [95% CI, 0.41–1.24]; *p* = 0.226) (Table 4; Figure 2C) also showed no statistical significance among Medicaid‐insured and Commercially insured patients. The corresponding median OS for the IPW method was 51.9 months (95% CI, 19.1–NA) for Medicaid insured and 72.1 months (95% CI, 62.5–83.9) for Commercially insured, respectively (Figure 2C). For the unadjusted univariable analyses, see Figure S1B,C.

#### 3.3.2 Time to second‐line therapy or death due to any cause

**FIGURE 3:**
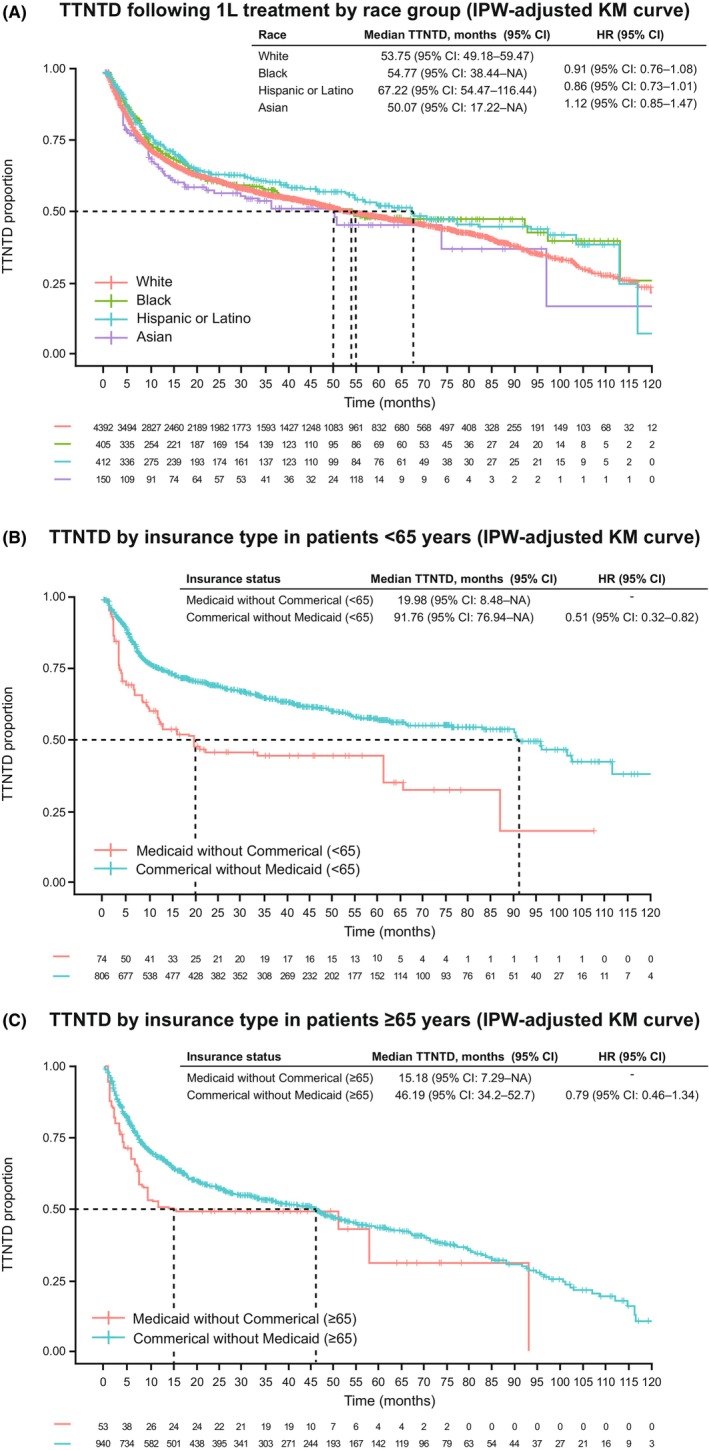
(A) TTNTD following 1 L treatment (IPW‐adjusted KM curve) by race group. (B) TTNTD by insurance type in patients aged <65 years. (C) TTNTD by insurance type in patients aged ≥65 years. 1 L, first‐line; CI, confidence interval; HR, hazard ratio; IPW, inverse probability weighting; KM, Kaplan–Meier; NA, not available; TTNTD, time to second‐line treatment or death.

After adjusting for statistically significant variables associated with TTNTD in the univariable analysis (Appendix S1 and Table S2), multivariable analyses, based on the Cox model (*p* = 0.099) and IPW method (*p* = 0.896), did not show statistically significant differences in TTNTD across race groups. The corresponding HRs (Black, Hispanic or Latino, and Asian vs. White patients) were 0.89 (95% CI, 0.75–1.05), 0.85 (95% CI, 0.73–1.00), and 1.11 (95% CI, 0.86–1.43), in multivariable analyses based on the Cox model (Table 4), and 0.91 (95% CI, 0.76–1.08), 0.86 (95% CI, 0.73–1.01), and 1.12 (95% CI, 0.85–1.47), for the IPW method (Figure 3A). Median TTNTD values for the IPW method were 54.8 months (95% CI, 38.4–NA) in Black patients, 67.2 months (95% CI, 54.5–116.4) in Hispanic or Latino patients, 50.1 months (95% CI, 17.2–NA) in Asian patients, and 53.8 months (95% CI, 49.2–59.5) in White patients (Figure 3A). For the unadjusted univariable analyses, see Figure S2A.

For patients aged <65 years, multivariable analysis based on the Cox model (HR, 0.70 [95% CI, 0.49–0.99]; *p* = 0.044) and IPW method (HR, 0.51 [95% CI, 0.32–0.82]; *p* = 0.005) showed statistically significantly shorter TTNTD among Medicaid‐insured patients than in Commercially insured patients (Table 4; Figure 3B). Median TTNTD by the IPW method was 20.0 months (95% CI, 8.5–NA) for Medicaid‐insured and 91.8 months (95% CI, 76.9–NA) for Commercially insured patients (Figure 3B). Among patients aged ≥65 years, TTNTD was not significantly different in Medicaid‐insured versus Commercially insured patients based on the Cox model (HR, 0.77 [95% CI, 0.53–1.12]; *p* = 0.172) or IPW analyses (HR, 0.79 [95% CI, 0.46–1.34]; *p* = 0.379) (Table 4; Figure 3C). The corresponding median TTNTD for the IPW method was 15.2 months (95% CI, 7.3–NA) for Medicaid‐insured and 46.2 months (95% CI, 34.2–52.7) for Commercially insured patients, respectively (Figure 3C). For the unadjusted univariable analyses, see Figure S2B,C.

## 4 DISCUSSION

In this analysis of data from the Flatiron Health database, which included over 5300 patients with DLBCL treated in the real world, OS was similar among patients of White, Black, Hispanic or Latino, and Asian race after adjusting for baseline characteristics. However, we found that among patients aged <65 years, using Medicaid without access to Commercial insurance was associated with worse OS and a shorter TTNTD when compared with Commercially insured patients.

In the current study, after adjusting for baseline characteristics, race was not a significant factor associated with survival outcomes in patients with DLBCL. This contradicts the findings of some early studies in the literature, which reported worse survival outcomes in Black patients with DLBCL compared with White patients.^17^, ^22^ However, recent data from large single‐center studies have shown similar survival outcomes in Black patients compared with White patients,^19^ and even improved survival in Hispanic or Latino patients compared with non‐Hispanic White patients with DLBCL.^18^ A retrospective analysis by Flowers et al, 2013^23^ suggested that the discrepancy in racial disparity observed between early studies and more recent studies could have arisen from the change in treatment regimen since January 2011, from predominantly using CHOP to R‐CHOP. Racial disparity was no longer shown in patients with DLBCL treated with R‐CHOP. The Flatiron database only included patients from 2011 onwards and ~ 90% of patients with DLBCL were treated with R‐CHOP.

Our finding that insurance status (Medicaid‐insured) was strongly associated with shorter survival outcomes in patients aged <65 years treated for DLBCL is consistent with previously published findings. In a report based on data from the NCDB, patients with DLBCL aged 18–64 years using Medicaid had inferior survival (HR for death, 1.48 [95% CI, 1.23–1.78]) compared with privately insured patients.^15^ Similarly, adjusted data from the SEER database showed that survival estimates for Medicaid‐insured patients with DLBCL were statistically significantly lower compared with non‐Medicaid‐insured patients; 3‐year cause‐specific survival estimates were 75% for patients with Medicaid and 89% for patients with other insurance.^16^ Patients aged <65 years with Medicaid insurance were diagnosed with more advanced disease (stage III–IV) when compared with patients with commercial insurance (without Medicaid) (65.8% vs. 47.7%; *p* = 0.005). This difference was not observed in patients aged ≥65 years (*p* = 0.400). Broadening access to affordable health insurance at an early point in the diagnostic process could reduce the survival disadvantage for patients without adequate coverage.

In addition to survival, our analysis reported TTNTD (which was lacking in prior work) and found that Medicaid‐insured patients aged <65 years had shorter TTNTD when compared with Commercially insured patients (20.0 months vs. 91.8 months, respectively). Both disease‐specific (i.e., later stage at diagnosis) and DLBCL‐unrelated factors (i.e., unfavorable socioeconomic status and related access barriers) likely contributed to the differences observed according to insurance status.

Similar to other studies in the literature, our data also show that Black patients with DLBCL, when compared with White patients, were diagnosed at a younger age^24^ and male gender was associated with statistically significantly worse OS and TTNTD.^15^

Using real‐world data has its own uniqueness and strengths. The Flatiron Health database includes comprehensive patient information with long follow up. However, limitations also exist, including missingness of some variables; only around 25% of the insurance data in the Flatiron Health database included both insurance start and end dates (though a missing end date may mean the insurance is valid, without yet reaching its termination date).Another potential limitation was the lack of database information on confounders of survival such as patient comorbidity score and supportive care, preventing their inclusion as covariates in multivariable analyses. Finally, although our cohort was quite large, the number of Medicaid patients was relatively small compared with Commercial patients, suggesting that there is scope for further large‐scale studies to be conducted in these populations. While race was not independently associated with long‐term DLBCL outcomes, Black patients were much more likely to have Medicaid insurance.

In conclusion, real‐world data from the USA nationwide Flatiron Health database suggest that race is not independently associated with survival outcomes in patients with DLBCL. Instead, insurance status (Medicaid‐insured) was significantly associated with shorter survival outcomes among patients aged <65 years but not in those aged ≥65 years. The median OS and TTNTD observed in these real‐world data were broadly aligned with previous clinical trials in patients with DLBCL.^7^ Future studies are needed to further evaluate the relationship between race/ethnicity and insurance status and the impact on survival outcomes in patients with DLBCL, and should consider social, environmental, biologic, and patient‐related factors that contribute to potential disparities in DLBCL survival.

## AUTHOR CONTRIBUTIONS

**Yanling Jin:** Conceptualization (equal); data curation (equal); formal analysis (equal); investigation (equal); writing – review and editing (equal). **Jia Li:** Investigation (equal); writing – review and editing (equal). **Yong Mun:** Conceptualization (equal); data curation (equal); formal analysis (equal); investigation (equal); writing – review and editing (equal). **Anthony Masaquel:** Investigation (equal); writing – review and editing (equal). **Sylvia Hu:** Conceptualization (equal); data curation (equal); formal analysis (equal); investigation (equal); writing – review and editing (equal). **Juliana M. L. Biondo:** Investigation (equal); writing – review and editing (equal).

## FUNDING INFORMATION

This study was sponsored by Genentech, Inc.

## CONFLICT OF INTEREST STATEMENT

YJ, JL, YM, AM, and JB: current employment, and current equity holder in a publicly traded company (Genentech Inc./F. Hoffmann‐La Roche Ltd). SH: previous employment, and previous equity holder in a publicly traded company (F. Hoffmann‐La Roche Ltd).

## Supporting information


Data S1.

Appendix S1.

Figure S1.

Figure S2.

Table S1.

## Data Availability

For up‐to‐date details on Roche's Global Policy on the Sharing of Clinical Information and how to request access to related clinical study documents, see here: https://go.roche.com/data_sharing. The de‐identified data that support the findings of this study are subject to a license agreement with Flatiron Health; interested researchers should contact dataaccess@flatiron.com to determine licensing terms. Anonymized records for individual patients across more than one data source external to Roche cannot, and should not, be linked due to a potential increase in risk of patient re‐identification.
