# Supplementary material for: Association of race/ethnicity and insurance with survival in patients with diffuse large B‐cell lymphoma in a large real‐world cohort
Source: Cancer Med. 2024 Aug 23;13(16):e70032. doi: 10.1002/cam4.70032 (PMC11342043; doi:10.1002/cam4.70032)
Supplement: Supplementary file 1 — Data S1. [file CAM4-13-e70032-s005.docx]

SUPPLEMENTARY FIGURES LEGENDS

SUPPLEMENTARY FIGURE 1 OS from start of treatment (unadjusted KM curve) by race group (A); and by insurance type in patients aged <65 years (B); and patients aged ≥65 years (C)

CI, confidence interval; HR, hazard ratio; KM, Kaplan-Meier; NA, not available; OS, overall survival.

SUPPLEMENTARY FIGURE 2 TTNTD following 1L treatment (unadjusted KM curve) by race group (A); and TTNTD by insurance type in patients aged <65 years (B); and patients aged ≥65 years (C)

CI, confidence interval; HR, hazard ratio; KM, Kaplan-Meier; NA, not available; TTNTD, time to second-line treatment or death.
