# Supplementary material for: Association of race/ethnicity and insurance with survival in patients with diffuse large B‐cell lymphoma in a large real‐world cohort
Source: Cancer Med. 2024 Aug 23;13(16):e70032. doi: 10.1002/cam4.70032 (PMC11342043; doi:10.1002/cam4.70032)
Supplement: Supplementary file 2 — Appendix S1. [file CAM4-13-e70032-s002.docx]

**Supplementary Appendix**

RESULTS

Time-to-event endpoints

Overall survival

*Univariable analysis*

Overall survival (OS) was statistically significantly different across race groups based on univariable analysis (*P* = 0.002). Median OS (unadjusted) was 112.6 months (95% confidence interval [CI], 97.0–not available [NA]) for Black patients, 112.5 months (95% CI, 96.9–NA) for Hispanic or Latino patients, NA (95% CI, 96.6–NA) for Asian patients, and 88.3 months (95% CI, 84.2–93.0) for White patients (Suppl Figure 1A); respective hazard ratios (HRs) were 0.76 [95% CI, 0.63–0.93], 0.79 [95% CI, 0.65–0.95] and 0.73 [95% CI, 0.52–1.02], vs. White patients (Suppl Table 2; Suppl Figure 1A).

In patients aged <65 years, median OS was significantly shorter in Medicaid-insured patients than in those with Commercial insurance (74.4 months [95% CI, 61.8–NA] vs. NA [95% CI, 112.5–NA]; HR, 0.48 [95% CI, 0.31–0.73; *P* = 0.001; Suppl Figure 1B). Among patients aged ≥65 years, median OS was similar in Medicaid and Commercially-insured patients (67.1 months [95% CI, 30.7–NA] vs. 72.1 months [95% CI, 62.5–83.0]; HR, 0.85 [95% CI, 0.55–1.29]; *P* = 0.435; Suppl Figure 1C).

Patient age at first-line treatment, sex, Eastern Cooperative Oncology Group (ECOG) performance status at initial diagnosis, disease stage at initial diagnosis, extranodal disease status, cell of origin, *BCL2*, *BCL6*, *CD30*, MYC, lactate dehydrogenase [LDH] level, site of care, and insurance status were also statistically significantly associated with OS (Suppl Table 2).

Time to second-line treatment or death due to any cause

*Univariable analysis*

Time to second-line therapy or death due to any cause (TTNTD) was statistically significantly different across race groups based on univariable analysis (*P* = 0.018) (Suppl Table 2). Median TTNTD (unadjusted) was 52.0 months (95% CI, 48.5–58.1) for White patients, 66.2 months (95% CI, 47.6–NA) for Black patients, 70.3 months (95% CI, 56.9–NA) for Hispanic or Latino patients, and 50.7 months (95% CI, 29.7–NA) for Asian (HR, 0.85 [95% CI, 0.72–1.0], 0.81 [95% CI, 0.69–0.95] and 1.01 [95% CI, 0.79–1.3], respectively, vs. White patients) (Suppl Figure 2A).

In patients aged <65 years, median TTNTD in Medicaid-insured patients was significantly shorter than in Commercially insured patients (34.0 months [95% CI, 13.0–NA] vs. 91.8 months [95% CI, 83.8–NA]; HR, 0.62 [95% CI, 0.44–0.88; *P* = 0.007) (Suppl Table 2; Suppl Figure 2B). Among patients aged ≥65 years, median TTNTD was not significantly different between Medicaid and Commercially insured patients (15.7 months [95% CI, 8.6–NA] vs. 45.8 months [95% CI, 33.7–51.7]) (Suppl Figure 2C).

Patient age at first-line treatment, gender, ECOG performance status at initial diagnosis, disease stage at initial diagnosis, transformation status, extranodal disease, cell of origin, *BCL2*, *BCL6*, LDH level, site of care, and insurance status were all statistically significantly associated with TTNTD (Suppl Table 2).
