# Supplementary figures and images for: Association of race/ethnicity and insurance with survival in patients with diffuse large B‐cell lymphoma in a large real‐world cohort
Source: Cancer Med. 2024 Aug 23;13(16):e70032. doi: 10.1002/cam4.70032 (PMC11342043; doi:10.1002/cam4.70032)

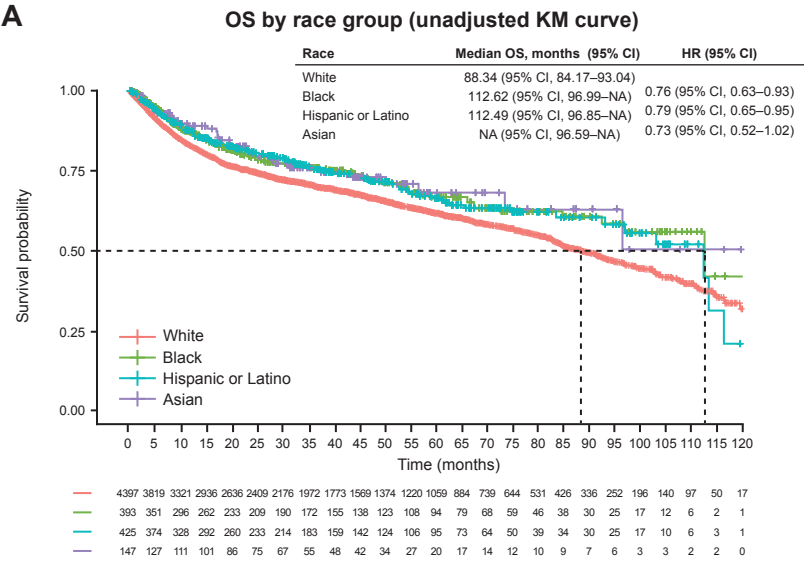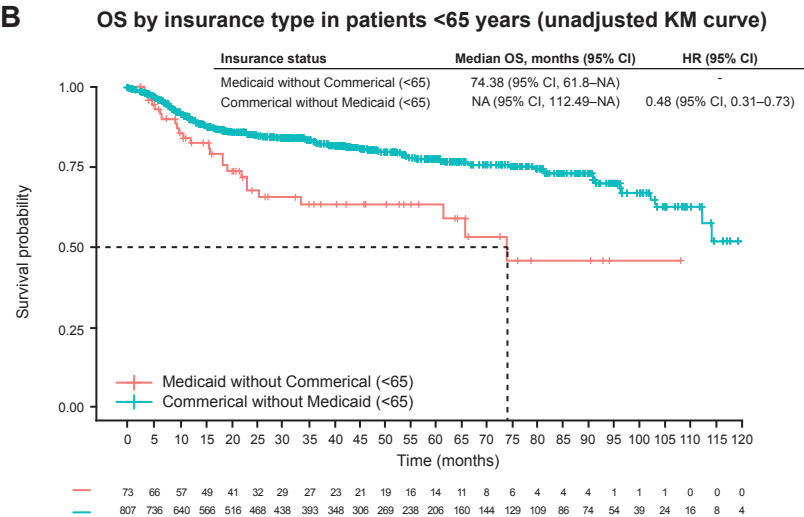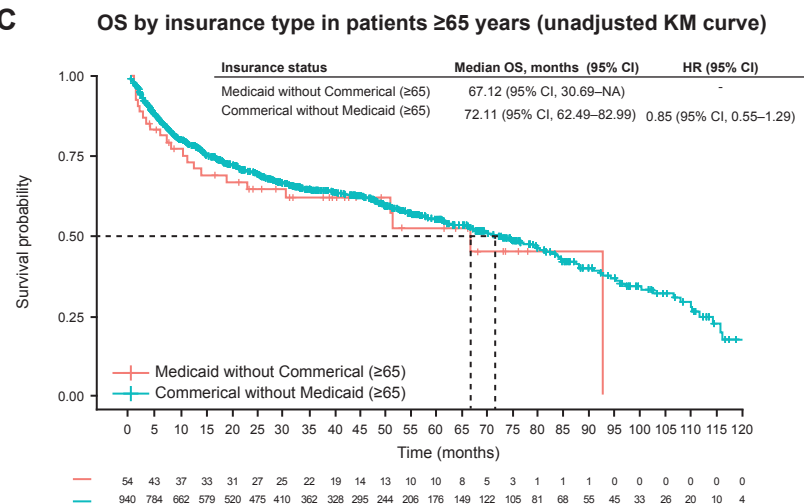

Supplement: Supplementary file 3 — Figure S1. [file CAM4-13-e70032-s001.pdf]
