# Supplementary material for: Association of race/ethnicity and insurance with survival in patients with diffuse large B‐cell lymphoma in a large real‐world cohort
Source: Cancer Med. 2024 Aug 23;13(16):e70032. doi: 10.1002/cam4.70032 (PMC11342043; doi:10.1002/cam4.70032)
Supplement: Supplementary file 4 — Figure S2. [file CAM4-13-e70032-s004.pdf]

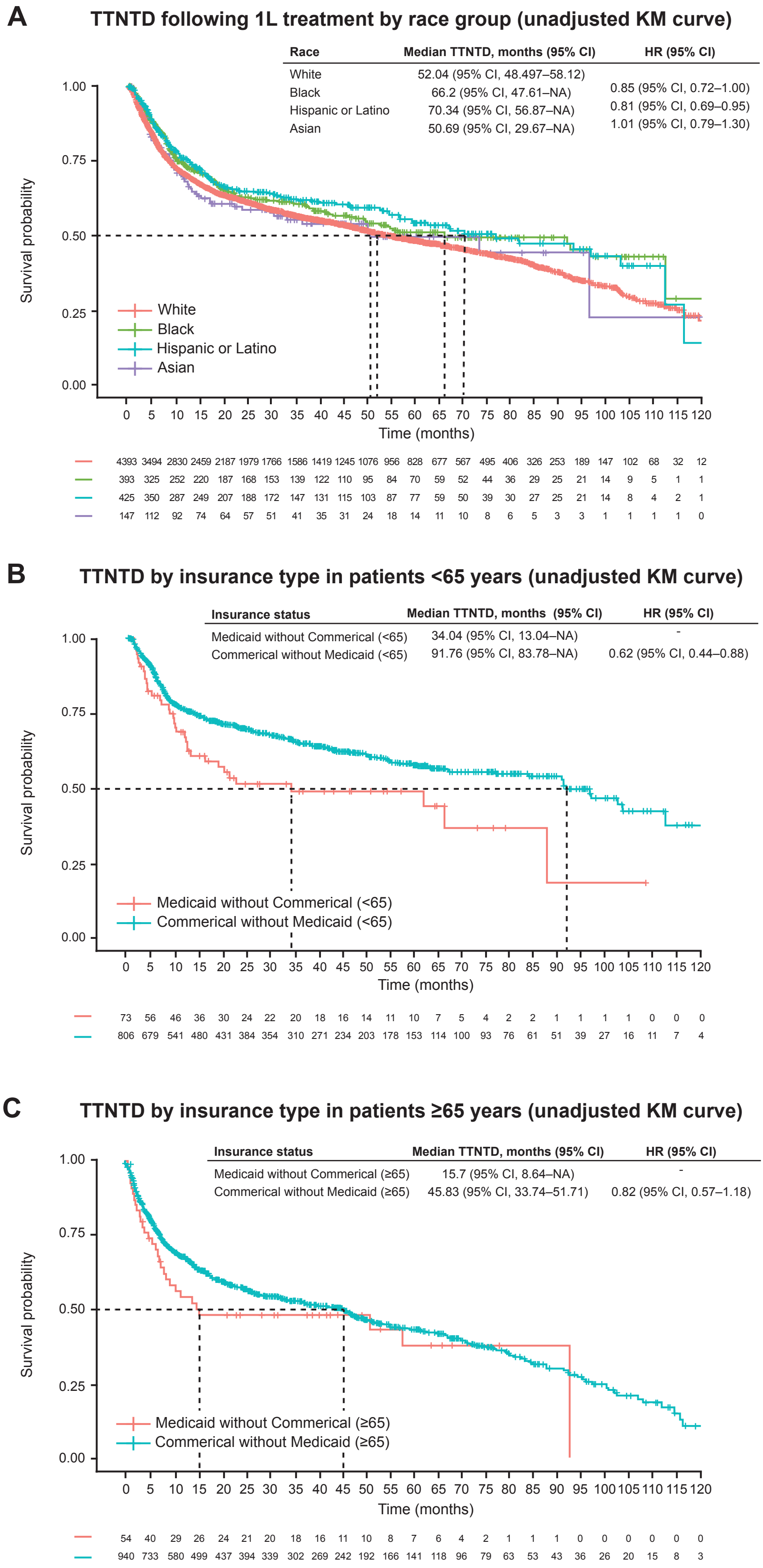
A
TTNTD following 1L treatment by race group (unadjusted KM curve)
Race | Median TTNTD, months (95% CI) | HR (95% CI)
White | 52.04 (95% CI, 48.497–58.12)
Black | 66.2 (95% CI, 47.61–NA) | 0.85 (95% CI, 0.72–1.00)
Hispanic or Latino | 70.34 (95% CI, 56.87–NA) | 0.81 (95% CI, 0.69–0.95)
Asian | 50.69 (95% CI, 29.67–NA) | 1.01 (95% CI, 0.79–1.30)
Survival probability
1.00
0.75
0.50
0.25
0.00
White
Black
Hispanic or Latino
Asian
0 5 10 15 20 25 30 35 40 45 50 55 60 65 70 75 80 85 90 95 100 105 110 115 120
Time (months)
4393 3494 2830 2459 2187 1979 1766 1586 1419 1245 1076 956 828 677 567 495 406 326 253 189 147 102 68 32 12
393 325 252 220 187 168 153 139 122 110 95 84 70 59 52 44 36 29 25 21 14 9 5 1 1
425 350 287 249 207 188 172 147 131 115 103 87 77 59 50 39 30 27 25 21 14 8 4 2 1
147 112 92 74 64 57 51 41 35 31 24 18 14 11 10 8 6 5 3 3 1 1 1 1 0
B
TTNTD by insurance type in patients <65 years (unadjusted KM curve)
Insurance status | Median TTNTD, months (95% CI) | HR (95% CI)
Medicaid without Commerical (<65) | 34.04 (95% CI, 13.04–NA) | -
Commerical without Medicaid (<65) | 91.76 (95% CI, 83.78–NA) | 0.62 (95% CI, 0.44–0.88)
Survival probability
1.00
0.75
0.50
0.25
0.00
Medicaid without Commerical (<65)
Commerical without Medicaid (<65)
0 5 10 15 20 25 30 35 40 45 50 55 60 65 70 75 80 85 90 95 100 105 110 115 120
Time (months)
73 56 46 36 30 24 22 20 18 16 14 11 10 7 5 4 2 2 1 1 1 1 0 0 0
806 679 541 480 431 384 354 310 271 234 203 178 153 114 100 93 76 61 51 39 27 16 11 7 4
C
TTNTD by insurance type in patients ≥65 years (unadjusted KM curve)
Insurance status | Median TTNTD, months (95% CI) | HR (95% CI)
Medicaid without Commerical (≥65) | 15.7 (95% CI, 8.64–NA) | -
Commerical without Medicaid (≥65) | 45.83 (95% CI, 33.74–51.71) | 0.82 (95% CI, 0.57–1.18)
Survival probability
1.00
0.75
0.50
0.25
0.00
Medicaid without Commerical (≥65)
Commerical without Medicaid (≥65)
0 5 10 15 20 25 30 35 40 45 50 55 60 65 70 75 80 85 90 95 100 105 110 115 120
Time (months)
54 40 29 26 24 21 20 18 16 11 10 8 7 6 4 2 1 1 1 0 0 0 0 0 0
940 733 580 499 437 394 339 302 269 242 192 166 141 118 96 79 63 53 43 36 26 20 15 8 3
