# Supplementary material for: Association of race/ethnicity and insurance with survival in patients with diffuse large B‐cell lymphoma in a large real‐world cohort
Source: Cancer Med. 2024 Aug 23;13(16):e70032. doi: 10.1002/cam4.70032 (PMC11342043; doi:10.1002/cam4.70032)
Supplement: Supplementary file 5 — Table S1. [file CAM4-13-e70032-s003.docx]

SUPPLEMENTARY TABLES

SUPPLEMENTARY TABLE 1 Distribution of patients according to timing of treatment

|  | **Treatment within 90 days** | **Treatment after 90 days** |
| --- | --- | --- |
| Race |  |  |
| White (*n* = 4560) | 4,397 (96.4%) | 163 (3.6%) |
| Black (*n* = 418) | 393 (94.0%) | 25 (6.0%) |
| Hispanic or Latino (*n* = 451) | 425 (94.2%) | 26 (5.8%) |
| Asian (*n* = 155) | 147 (94.8%) | 8 (5.2%) |
| Insurance type |  |  |
| Medicaid (without Commercial) (*n* = 129) | 127 (98.4%) | 2 (1.6%) |
| Commercial (without Medicaid) (*n* = 418) | 1,747 (97.1%) | 53 (2.9%) |

SUPPLEMENTARY TABLE 2 Summary of time-to-event endpoints based on a Cox model (univariable analysis)

|  | **Univariable** | | |
| --- | --- | --- | --- |
|  | **HR (95% CI)** | **Pair-wise  *P*** | **Overall *P*** |
| **Overall survival** |  |  |  |
| Race |  |  |  |
| White (ref) |  |  |  |
| Black | 0.76 (0.63–0.93) | 0.008 | 0.002 |
| Hispanic or Latino | 0.79 (0.65–0.95) | 0.014 |  |
| Asian | 0.73 (0.52–1.02) | 0.068 |  |
| Age* |  |  |  |
| <50 years (ref) |  |  |  |
| 50–64 years | 1.96 (1.53–2.51) | < 0.001 | < 0.001 |
| 65–79 years | 3.98 (3.16–5.02) | < 0.001 |  |
| ≥80 years | 6.33 (4.87–8.24) | < 0.001 |  |
| Sex |  |  |  |
| Female (ref) |  |  |  |
| Male | 1.16 (1.06–1.28) | 0.002 | 0.002 |
| ECOG PS |  |  |  |
| 0–1 (ref) |  |  |  |
| ≥2 | 2.65 (2.25–3.11) | < 0.001 | < 0.001 |
| Missing | 1.07 (0.96–1.18) | 0.229 |  |
| Disease stage^†^ |  |  |  |
| I–II (ref) |  |  |  |
| III–IV | 1.75 (1.54–1.97) | < 0.001 | < 0.001 |
| Missing | 2.33 (2.03–2.67) | < 0.001 |  |
| Transformed from a prior indolent lymphoid malignancy |  |  |  |
| Yes (ref) |  |  |  |
| No/unknown | 1.06 (0.93–1.21) | 0.357 | 0.357 |
| Extranodal disease at diagnosis |  |  |  |
| Yes (ref) |  |  |  |
| No/unknown | 1.25 (1.14–1.38) | < 0.001 | < 0.001 |
| Cell of origin^†^ |  |  |  |
| GCB (ref) |  |  |  |
| ABC | 1.24 (1.08–1.42) | 0.002 | 0.005 |
| Unknown/undocumented | 1.06 (0.95–1.18) | 0.333 |  |
| BCL2 |  |  |  |
| Positive (ref) |  |  |  |
| Negative | 0.71 (0.61–0.83) | < 0.001 | < 0.001 |
| Unknown | 0.91 (0.83–1.01) | 0.080 |  |
| BCL6 |  |  |  |
| Positive (ref) |  |  |  |
| Negative | 1.25 (1.07–1.47) | 0.005 | 0.014 |
| Unknown | 1.08 (0.98–1.19) | 0.136 |  |
| CD30 |  |  |  |
| Positive (ref) |  |  |  |
| Negative | 1.27 (1.06–1.53) | 0.010 | 0.021 |
| Unknown | 1.27 (1.07–1.51) | 0.007 |  |
| EBER |  |  |  |
| Positive (ref) |  |  |  |
| Negative | 0.84 (0.50–1.39) | 0.491 | 0.720 |
| Unknown | 0.87 (0.53–1.43) | 0.592 |  |
| MYC^#^ |  |  |  |
| Positive (ref) |  |  |  |
| Negative | 0.77 (0.61–0.95) | 0.018 | 0.035 |
| Unknown | 0.86 (0.75–0.99) | 0.033 |  |
| Elevated LDH |  |  |  |
| No (ref) |  |  |  |
| Yes | 1.50 (1.31–1.73) | < 0.001 | < 0.001 |
| Unknown | 1.36 (1.18–1.56) | < 0.001 |  |
| Site of care |  |  |  |
| Academic (ref) |  |  |  |
| Community | 1.21 (1.05–1.39) | 0.007 | 0.007 |
| Insurance type |  |  |  |
| <65 years: Medicaid (without Commercial) |  |  |  |
| <65 years: Commercial (without Medicaid) | 0.48 (0.31–0.73) | 0.001 | 0.001 |
| <65 years: Other | 0.44 (0.29–0.67) | < 0.001 |  |
| ≥65 years: Medicaid (without Commercial) |  |  |  |
| ≥65 years: Commercial (without Medicaid) | 0.86 (0.57–1.31) | 0.492 | 0.731 |
| ≥65 years: Other | 0.89 (0.59–1.35) | 0.583 |  |
| **Time to second-line therapy or death due to any cause** |  |  |  |
| Race |  |  |  |
| White (ref) |  |  |  |
| Black | 0.85 (0.72–1.00) | 0.044 | 0.018 |
| Hispanic or Latino | 0.81 (0.69–0.95) | 0.010 |  |
| Asian | 1.01 (0.79–1.3) | 0.932 |  |
| Age* |  |  |  |
| <50 years (ref) |  |  |  |
| 50–64 years | 1.34 (1.14–1.58) | < 0.001 | < 0.001 |
| 65–79 years | 1.95 (1.68–2.27) | < 0.001 |  |
| ≥80 years | 2.65 (2.20–3.20) | < 0.001 |  |
| Sex |  |  |  |
| Female (ref) |  |  |  |
| Male | 1.12 (1.03–1.21) | 0.005 | 0.005 |
| ECOG PS |  |  |  |
| 0–1 (ref) |  |  |  |
| ≥2 | 2.09 (1.80–2.41) | < 0.001 | < 0.001 |
| Missing | 1.07 (0.98–1.16) | 0.137 |  |
| Group Stage status^†^ |  |  |  |
| I–II (ref) |  |  |  |
| III–IV | 1.78 (1.61–1.97) | < 0.001 | < 0.001 |
| Missing | 2.18 (1.94–2.45) | < 0.001 |  |
| Transformed from a prior indolent lymphoid malignancy |  |  |  |
| Yes (ref) |  |  |  |
| No/unknown | 1.34 (1.21–1.49) | < 0.001 | < 0.001 |
| Extranodal disease involvement at initial diagnosis |  |  |  |
| Yes (ref) |  |  |  |
| No/unknown | 1.22 (1.12–1.32) | < 0.001 | < 0.001 |
| Cell of origin^†^ |  |  |  |
| GCB (ref) |  |  |  |
| ABC | 1.16 (1.03–1.29) | 0.011 | 0.014 |
| Unknown/undocumented | 1.01 (0.92–1.10) | 0.904 |  |
| BCL2 |  |  |  |
| Positive (ref) |  |  |  |
| Negative | 0.68 (0.60–0.78) | < 0.001 | < 0.001 |
| Unknown | 0.87 (0.8–0.94) | 0.001 |  |
| BCL6 |  |  |  |
| Positive (ref) |  |  |  |
| Negative | 1.24 (1.08–1.41) | 0.002 | 0.006 |
| Unknown | 1.00 (0.92–1.09) | 0.956 |  |
| CD30 |  |  |  |
| Positive (ref) |  |  |  |
| Negative | 0.97 (0.84–1.12) | 0.685 | 0.673 |
| Unknown | 1.01 (0.88–1.16) | 0.874 |  |
| EBER |  |  |  |
| Positive (ref) |  |  |  |
| Negative | 0.68 (0.45–1.02) | 0.064 | 0.157 |
| Unknown | 0.68 (0.46–1.01) | 0.056 |  |
| MYC^#^ |  |  |  |
| Positive (ref) |  |  |  |
| Negative | 0.89 (0.75–1.06) | 0.200 | 0.081 |
| Unknown | 0.88 (0.78–0.98) | 0.025 |  |
| Elevated LDH |  |  |  |
| No (ref) |  |  |  |
| Yes | 1.58 (1.40–1.77) | < 0.001 | < 0.001 |
| Unknown | 1.46 (1.30–1.64) | < 0.001 |  |
| Site of care |  |  |  |
| Academic (ref) |  |  |  |
| Community | 0.89 (0.8–0.99) | 0.026 | 0.026 |
| Insurance type |  |  |  |
| <65 years: Medicaid (without Commercial) |  |  |  |
| <65 years: Commercial (without Medicaid) | 0.61 (0.43–0.87) | 0.006 | 0.009 |
| <65 years: Other | 0.59 (0.42–0.83) | 0.002 |  |
| ≥65 years: Medicaid (without Commercial) |  |  |  |
| ≥65 years: Commercial (without Medicaid) | 0.83 (0.58–1.20) | 0.332 | 0.583 |
| ≥65 years: Other | 0.86 (0.60–1.23) | 0.411 |  |

Data are HR (95% CI). OS was defined as time from start of the first line treatment to death or censor at last alive date. TTNTD was defined as time from the start of the first line treatment. Please note, ‘ref’ refers to the reference group for the p-value comparison for each variable.

Abbreviations: ABC, non-germinal B-cell/activated B-cell; CI, confidence interval; EBER, Epstein-Barr encoded RNA; ECOG PS, Eastern Cooperative Oncology Group performance status; GCB, germinal center B-cell; HR, hazard ratio; IHC, immunohistochemistry; LDH, lactate dehydrogenase.

^*^At first treatment.

^†^At initial diagnosis.

^#^By IHC.
